# Prevalence of malnutrition among children at primary cleft surgery: A cross-sectional analysis of a global database

**DOI:** 10.7189/jogh.12.04012

**Published:** 2022-02-26

**Authors:** Barbara Delage, Erin Stieber, Pamela Sheeran

**Affiliations:** 1 London School of Hygiene and Tropical Medicine, London, UK; 2 Smile Train, New York City, New York, USA

## Abstract

**Background:**

Orofacial clefts are common birth defects, affecting one newborn in every 700 live births globally. The condition requires prompt identification, feeding support, and timely surgery. While orofacial clefts benefit from a comprehensive, life-long care management in high-income countries, care provision is often lacking or inadequate in poor-resource settings. Data on the burden of orofacial clefts in children born in limited-resource settings remain scarce. The objective of this study was to estimate the prevalence of malnutrition in children using cleft surgery records collected by one large non-governmental cleft organization in low- and middle-income countries (LMICs).

**Methods:**

The data set included clinical records of children ≤5 years who underwent primary cleft surgery between 2008 and 2018 in health care facilities across LMICs. Patients’ data included birth date, sex, weight at surgery, ethnicity, country of origin, and date of primary surgery and were analysed using descriptive statistics. The prevalence of malnutrition was estimated using weight-for-age z scores and the distribution described in relation to cleft type, sex, ethnic groups, and geography. Comparisons with prevalence estimates for underweight in children under-5 within countries were conducted using publicly available survey data.

**Results:**

The analysis included 602 568 children. The prevalence of underweight in children with cleft varies with the epidemiology of cleft and the timing of primary surgery, and between ethnic groups and settings. The overall prevalence of underweight at the time of primary cleft surgery was 28.6% – a figure well above the global underweight prevalence in children under-5 without cleft estimated at 13.5%. We found a positive correlation between the prevalence of underweight among children with cleft and the prevalence of underweight in the DHS program (r_s_ = 0.6305; *P* < 0.0001). Within-country comparisons showed that, with only few exceptions, the prevalence of underweight was higher in children with clefts than in their peers born without clefts (*P* < 0.05).

**Conclusions:**

Although orofacial cleft is not considered to be a life-threatening condition, our findings show a high burden of malnutrition among patients accessing surgeries in LMICs. Interventions prompting early identification and appropriate feeding management of this group of vulnerable children is essential to leave no one behind in the fight against malnutrition.

There is substantial global disparity in the care of orofacial cleft, a treatable condition presenting in one in 700 newborns. A comprehensive approach to cleft care is needed and should involve a range of specialist health workers including lactation specialists, nutritionists, paediatricians, anaesthesiologists, cleft surgeons, ENT specialists, dentists, orthodontists, and speech therapists [1]. In high-income countries, perinatal nursing care ensures an early diagnosis and the monitoring of any feeding and/or breathing issues that could interfere with growth and development. In contrast, in resource-constrained settings where the coverage of essential maternal and child health services is low [2], the need for early cleft identification and comprehensive assessment and care of the patients is not easily met, imposing a major burden to patients and their families. If the survival of newborns with cleft may be at stake, especially in settings of widespread malnutrition and high disease burden, only a handful of small studies attempted to report on the situation [3,4]. To expand the evidence base and encourage further international efforts towards leaving no one behind including children born with a cleft, we undertook the analysis of a global clinical database owned by the largest charitable cleft organisation to estimate the prevalence of underweight in children with cleft across low- and middle-income countries [5].

## METHODS

### Study design and participants

This cross-sectional study included records of individuals who underwent primary surgery for orofacial clefts in Smile Train-sponsored facilities in LMICs. Only information uploaded by local cleft care providers into Smile Train’s online medical database between July 1, 2008, and June 30, 2018, has been retrieved (N = 868 854 entries). The study was restricted to patients aged ≤5 years at the time of surgery (N = 638 988 entries). Reasons for this included the fact that (i) orofacial clefts can complicate the feeding of newborns and immediately place them at risk of malnutrition, (ii) WHO growth standards are available to assess the nutrition status of children ≤5 years old, and (iii) country-specific estimates on malnutrition in children aged ≤5 years are accessible from the Demographic and Health Survey (DHS) program for comparison.

### Variables

Deidentified records of patients included date of birth, sex, weight at surgery, ethnicity, country of origin, cleft type, and date of cleft operation. Age at surgery was calculated using date of birth and date of operation and any erroneous values (≤0 years) or values >5 years were excluded. Out of eight ethnic groups reported, Pacific islanders that represented a minority of cases (N = 53; <0.1%) were pooled with Asians and those identified as “mixed” and “other” were pooled together. For descriptive analyses, countries were grouped according to six world regions as defined by WHO. Information regarding cleft types in Smile Train’s clinical records included the anatomic location (lip, alveolus, hard palate, soft palate, submucous hard/soft palate), laterality (left, right, bilateral), and completeness (complete/incomplete) of the cleft. Cases with clefts of the hard and/or soft palate also included rarer cases of submucous clefts of the palate. Isolated alveolar clefts were categorized with clefts of the palate. Cleft types, whether of the lip, alveolus, or palate, were considered regardless of their completeness. We classified clefts according to three main categories (cleft lip only [CLO], cleft palate only [CPO], and cleft lip and palate [CLP]) according to ICD-10 [6]. Cases with missing information on any of the cleft anatomic locations and those erroneously reported as having no clefts were excluded from analyses. The weight-for-age z score anthropometric index was generated from weight, age, and sex variables, using WHO Stata macro [7]. The prevalence of underweight corresponded to the percentage of children with weight-for-age z scores<-2 SD away from the mean of the reference population [8]. Extreme weight-for-age z-score values were flagged as outliers according to WHO cleaning criteria ie, z scores either<-6 SD or>+5 SD. Patients with extreme values or missing z-scores due to missing or negative weight values were also excluded.

### Analyses

Data management and descriptive statistics were conducted using Stata/IC v14.2 (StataCorp, College Station TX, USA). We seldom used small-sample statistical inference [9]. Instead, we reported confidence intervals (CI) that provide a range of the magnitude of a variable of interest. We calculated the median age at surgery, the 25^th^ percentile (Q1), and the 75^th^ percentile (Q3). Primary surgery was considered late in children with CL±P over 1 year of age and in children with CPO over 2 years of age. For comparison with country-specific survey data, we arbitrarily excluded countries with a total number of cases <200 (41 countries and 2168 cases excluded) and examined the prevalence of underweight in the remaining 60 countries. The most recent estimates of underweight prevalence in children aged 0-5 years in 42 countries (21 African countries, 4 in the Eastern Mediterranean region, 3 in the European region, 7 in Latin America, 6 in South-East Asia, and 1 in the Western Pacific region) were extracted from the DHS program using STATcompiler [10]. At country level, the prevalence of underweight in children with cleft was compared to the national DHS estimate (assumed to be - for statistical purpose - the true population prevalence) using a Z-test at a significance level of 5%. Finally, the correlation between the prevalence of underweight in children with cleft and DHS estimates across countries was tested using a Spearman’s rank test.

### Ethics

Patients or their caregivers have consented to their data being captured and used by Smile Train for reviews of quality, education, evaluation, and for marketing and communication purposes. This study was approved on June 1, 2018, by the MSc Research Ethics Committee at the London School of Hygiene and Tropical Medicine (ref: 15433).

## RESULTS

Records from 638 988 children aged ≤5 years at the time of primary cleft surgery were retrieved from Smile Train’s database for the period between July 1, 2008, and June 30, 2018. Information on weight was missing for 4843 cases (0.8%) and erroneously reported for 556 cases (0.03%). Therefore, there was a total of 633 589 children for whom a weight-for-age z score could be calculated. A small percentage (3.6%) of z scores were flagged as extreme values and excluded, leaving 610 714 children for whom z-scores could be used to calculate the prevalence of underweight (z scores<-2 SD) in the data set. Among these children, 602 568 had valid information on cleft types.

### Epidemiological characteristics of children with orofacial clefts

Females represented about 40% of all cases (**Table 1**). Nearly half (49.1%) of the children were <1 year and 76.4% were <2 years of age. About half (48.8%) of the children were identified as Asians and 36% as Indians; other ethnic groups in the data set (15.2%) included Black Africans, Hispanics, Caucasians, and mixed /other groups. Children originated from 101 countries, grouped into six geographical regions (Table S1 in the **Online Supplementary Document**). Over 75% of the children in the data set were from South-East Asia and the Western Pacific region.

**Table 1:** Distribution of main cleft types in records of children ≤5 y in the clinical database

	Count	CLO (%)	CPO (%)	CLP (%)	Prevalence ratio CLO | CPO | CLP	Male-to-female ratio†		
						**CLO**	**CPO**	**CLP**
TOTAL*	602 568	19.2	19.3	61.5	1 | 1.0 | 3.2	1.12	0.64	1.12
**Sex:**								
Male	366 225	20.1	15.9	64.0	1 | 0.8 | 3.2	**–**	**–**	**–**
Female	236 343	17.9	24.7	57.4	1 | 1.4 | 3.2	**–**	**–**	**–**
**Age groups (months):**								
0-5	123 620	27.9	1.4	70.7	1 | 0.1 | 2.5	1.15	0.79	1.08
6-11	172 369	21.3	11.2	67.5	1 | 0.5 | 3.2	1.10	0.64	1.08
12-23	164 092	13.1	28.3	58.6	1 | 2.2 | 4.5	1.12	0.66	1.16
24-35	68 272	14.7	33.7	51.6	1 | 2.3 | 3.5	1.11	0.63	1.16
36-47	44 016	16.5	36.0	47.5	1 | 2.2 | 2.9	1.10	0.62	1.16
48-60	30 199	19.5	33.7	46.8	1 | 1.7 | 2.4	1.11	0.60	1.21
**Ethnic groups:**								
Asian	293 783	21.0	23.5	55.5	1 | 1.1 | 2.6	1.18	0.65	1.16
Black African	48 692	30.7	7.8	61.5	1 | 0.3 | 2.0	1.00	0.64	0.94
Indian	217 198	15.8	16.7	67.5	1 | 1.1 | 4.3	1.07	0.66	1.09
Hispanic	20 484	8.4	12.1	79.5	1 | 1.4 | 9.5	1.21	0.52	1.23
Caucasian	10 786	11.4	22.7	65.9	1 | 2.0 | 5.8	1.22	0.50	1.14
Mixed/Other	11 625	17.2	19.7	63.1	1 | 1.1 | 3.7	1.09	0.58	1.17
**World regions:**								
African Region	46 497	31.7	7.9	60.4	1 | 0.2 | 1.9	1.00	0.64	0.93
Eastern Mediterranean Region	45 052	15.0	20.2	64.8	1 | 1.3 | 4.3	1.13	0.63	1.06
European Region	6486	12.9	29.1	58.0	1 | 2.3 | 4.5	1.18	0.55	1.30
Region of the Americas	33 202	9.3	14.5	76.2	1 | 1.6 | 8.2	1.19	0.55	1.21
South-East Asia Region	285 627	16.3	15.4	68.3	1 | 0.9 | 4.2	1.07	0.66	1.08
Western Pacific Region	185 704	23.6	28.6	47.8	1 | 1.2 | 2.0	1.21	0.65	1.26

CLO – cleft lip only, CPO – cleft palate only, CLP – cleft lip and palate

*A total of 36 420 cases with either missing cleft/weight information or with missing/extreme z scores values have been removed from this analysis.

†Prevalence ratio.

Overall, three out of five children (61.5%) who underwent primary surgery had CLP, one in five had CLO (19.2%) and one in five had CPO (19.3%) (**Table 1**). Across all ethnic groups, over 50% of primary surgeries were in children with CLP, but the relative proportions of the three main cleft types varied with ethnicity and geography. The lowest CLP-to-CLO ratio (2:1) was found among Black Africans. In contrast, the number of primary surgeries was ten times higher in Hispanic children with CLP than in those with CLO. Further, only 7.9% of the primary surgeries in Africa were conducted in children with CPO (largely identified as Black Africans) whereas this group represented about 15% of all primary cleft surgeries in Latin America (primarily in Hispanics and Caucasians) and South-East Asia (primarily in Indians and Asians), 20% in the Eastern Mediterranean region (primarily in Asians and Indians), and close to 30% in the Western Pacific region (largely in Asians) and European region (primarily in Caucasians and Asians). We also observed sex differences in cleft types. Children with CPO were more likely to be females (sex prevalence ratio of 0.6) whereas there was a slight predominance of males among children with either CLO or CLP (sex prevalence ratio of 1.2). This pattern was observed across age groups, ethnic groups, and world regions (**Table 1**).

### Nutrition profile at primary surgery

Overall, 28.6% of children had a low weight-for-age z score (<-2 SD) at the time of surgery (**Table 2**). The prevalence of underweight varied with sex, age at surgery, ethnicity/geography, and type of cleft. Underweight at surgery was more prevalent among children with CLP (32.8% vs 25.5% with CLO and 18.4% with CPO), in males (30.9% vs 25.0% in females), in older children (33.6% at 48-60 months vs 26.9% at 0-5 months), and in children from South-East Asia (40.4%) – primarily Indians (43.7%). The prevalence of underweight at surgery was also systematically lower in children with CPO compared to those with CL ± P, regardless of sex, age at surgery, and ethnicity – except among Black Africans (**Table 2**).

**Table 2:** Prevalence of underweight at surgery according to the type of cleft and across sex, age groups, ethnic groups, and world regions

	% Weight-for-age Z score<-2 SD (95% CI)†			
	**CLO**	**CPO**	**CLP**	**Total clefts**
TOTAL*	25.5 (25.2-25.7)	18.4 (18.2-18.6)	32.8 (32.6-32.9)	28.6 (28.5-28.7)
**Sex:**				
Male	26.9 (26.6-27.2)	20.0 (19.7-20.4)	34.8 (34.6-35.0)	30.9 (30.7-31.0)
Female	23.0 (22.6-23.4)	16.8 (16.5-17.1)	29.2 (29.0-29.5)	25.0 (24.9-25.2)
**Age groups (months):**				
0-5	17.0 (16.6-17.4)	14.2 (12.6-15.9)	31.1 (30.8-31.4)	26.9 (26.7-27.2)
6-11	23.6 (23.2-24.1)	16.8 (16.3-17.3)	34.9 (34.6-35.1)	30.5 (30.2-30.7)
12-23	29.6 (29.0-30.2)	14.3 (14.0-14.6)	28.6 (28.3-28.9)	24.7 (24.5-24.9)
24-35	37.0 (36.1-37.9)	20.6 (20.0-21.1)	35.7 (35.2-36.2)	30.8 (30.4-31.1)
36-47	38.7 (37.6-39.9)	24.5 (23.8-25.2)	39.1 (38.5-39.8)	33.8 (33.4-34.2)
48-60	36.2 (34.9-37.4)	26.4 (25.5-27.3)	37.8 (37.0-38.6)	33.6 (33.1-34.2)
**Ethnic group:**				
Asian	15.8 (15.5-16.1)	11.4 (11.1-11.6)	23.0 (22.8-23.2)	18.7 (18.6-18.9)
Black African	24.3 (23.6-25.0)	24.8 (23.4-26.2)	32.6 (32.1-33.2)	29.5 (29.1-29.9)
Indian	44.3 (43.8-44.8)	32.5 (32.0-33.0)	46.3 (46.0-46.6)	43.7 (43.5-43.9)
Hispanic	17.4 (15.7-19.3)	13.3 (12.0-14.7)	21.5 (20.9-22.1)	20.2 (19.6-20.7)
Caucasian	17.3 (15.3-19.5)	10.0 (8.9-11.3)	15.4 (14.6-16.3)	14.4 (13.8-15.1)
Mixed/Other	22.7 (20.9-24.6)	12.2 (11.0-13.6)	22.6 (21.6-23.5)	20.6 (19.8-21.3)
**World region of origin:**				
African region	24.3 (23.6-25.0)	25.1 (23.7-26.5)	32.6 (32.0-33.1)	29.4 (28.9-29.8)
Eastern Mediterranean region	32.1 (31.0-33.2)	21.9 (21.1-22.8)	39.3 (15.0-17.5)	34.7 (34.2-35.1)
European region	11.9 (9.9-14.3)	8.0 (6.9-9.3)	12.4 (11.3-13.4)	11.0 (10.3-11.8)
Region of the Americas	16.2 (15.0-17.5)	11.9 (11.0-12.8)	18.5 (18.0-19.0)	17.3 (16.9-17.7)
South-East Asia region	39.9 (39.5-40.4)	31.6 (31.2-32.1)	42.5 (42.3-42.8)	40.4 (40.3-40.6)
Western Pacific region	10.4 (10.1-10.7)	7.4 (7.2-7.6)	14.2 (13.9-14.4)	11.3 (11.2-11.5)

CLO – cleft lip only, CPO – cleft palate only, CLP – cleft lip and palate, SD – standard deviation, CI – confidence interval

*602 568 cases.

†Percentage of underweight children.

Late primary surgery also contributed to a greater prevalence of underweight among children with CLO (33.6% vs 20.4% with timely surgery) and those with CPO (23.0% vs 15.0% with timely surgery), yet not among those with CLP for whom underweight is highly prevalent even among those operated before 1 year of age (33.2% with timely surgery vs 32.2% with late surgery) (**Table 3**). This pattern was observed with primary surgeries completed in Asians (CLO: 23.8% with late surgery vs 11.7% with timely surgery; CPO: 14.9% with late surgery vs 8.4% with timely surgery) and Indians (CLO: 50.3% with late surgery vs 39.4% with timely surgery; CPO: 43.5% with late surgery vs 26.8% with timely surgery), and consequently in the Western Pacific and South-East Asia Regions. The prevalence of underweight was also higher among Black Africans with CLO operated after 1 year of age (28.0% vs 20.8% with timely surgery) and Caucasians with CPO operated after 2 years of age (15.4% vs 7.9% with timely surgery). Median age at primary surgery and prevalence estimates of late primary surgeries across cleft types, sex, ethnic groups, and world regions are available as supplementary data (Table S2 in the **Online Supplementary Document**).

**Table 3:** Prevalence of underweight according to the timing of surgery. **‡**

	% Weight-for-age Z score<-2 SD (95% CI)					
	**CLO**		**CPO**		**CLP**	
	**Timely surgeries†**	**Late surgeries‡**	**Timely surgeries‡**	**Late surgeries‡**	**Timely surgeries†**	**Late surgeries‡**
TOTAL*	20.4 (20.1-20.7)	33.6 (33.2-34.0)	15.0 (14.7-15.3)	23.0 (22.7-23.4)	33.2 (33.0-33.5)	32.2 (32.0-32.4)
**Sex:**						
Male	22.6 (22.2-23.0)	33.9 (33.4-34.5)	17.2 (16.8-17.6)	24.0 (23.5-24.6)	36.8 (36.5-37.0)	32.5 (32.2-32.7)
Female	16.6 (16.2-17.1)	33.1 (32.4-33.8)	12.8 (12.4-13.1)	22.1 (21.6-22.6)	27.3 (27.0-27.7)	31.7 (31.3-32.0)
**Ethnic groups:**						
Asian	11.7 (11.4-12.1)	23.8 (23.2-24.4)	8.4 (8.1-8.7)	14.9 (14.5-15.3)	22.5 (22.2-22.8)	23.5 (23.2-23.8)
Black African	20.8 (20.0-21.8)	28.0 (27.0-29.1)	23.7 (22.0-25.6)	26.1 (24.1-28.3)	33.7 (33.1-34.5)	31.0 (30.2-31.9)
Indian	39.4 (38.7-40.1)	50.3 (49.5-51.1)	26.0 (25.5-26.6)	43.5 (42.6-44.3)	46.7 (46.3-47.0)	45.8 (45.4-46.2)
Hispanic	16.8 (14.8-18.9)	19.4 (15.8-23.4)	13.1 (11.5-14.9)	13.7 (11.6-16.1)	24.6 (23.8-25.5)	17.3 (16.4-18.2)
Caucasian	17.4 (15.1-19.9)	16.9 (12.7-22.1)	7.9 (6.7-9.3)	15.4 (12.9-18.3)	18.1 (16.9-19.3)	11.8 (10.7-13.0)
Mixed/Other	23.4 (21.2-25.7)	21.4 (18.5-24.7)	10.2 (8.8-11.8)	17.0 (14.3-20.0)	26.3 (25.1-27.6)	16.2 (14.9-17.7)
**World regions:**						
African Region	20.6 (19.7-21.5)	28.3 (27.2-29.3)	24.1 (22.3-26.0)	26.3 (24.2-28.6)	33.9 (33.2-34.6)	30.7 (29.8-31.5)
Eastern Mediterranean Region	35.4 (33.9-36.9)	27.5 (25.9-29.2)	17.3 (16.3-18.3)	29.4 (27.9-31.0)	43.2 (42.5-44.0)	33.9 (33.1-34.7)
European Region	11.8 (9.5-14.6)	10.3 (6.9-15.2)	6.2 (4.9-7.8)	10.5 (8.5-12.9)	13.2 (11.8-14.7)	11.1 (96.7-12.7)
Region of the Americas	16.6 (15.2-18.2)	14.8 (12.4-17.5)	10.7 (9.6-11.8)	14.2 (12.6-16.0)	21.6 (20.9-22.3)	14.3 (13.7-15.0)
South-East Asia Region	34.7 (34.1-35.3)	46.1 (45.4-46.7)	25.2 (24.7-25.8)	42.1 (41.4-42.9)	42.6 (42.3-42.9)	42.5 (42.1-42.8)
Western Pacific Region	7.4 (7.1-7.7)	18.0 (17.3-18.7)	4.7 (4.5-5.0)	10.3 (9.9-10.7)	12.9 (12.6-13.2)	15.6 (15.2-15.9)

CLO – cleft lip only, CPO – cleft palate only, CLP – cleft lip and palate, SD – standard deviation, CI – confidence interval

*602 568 cases.

†“Timely” corresponds to surgeries in children with CLO/CLP≤1 y of age; “late” corresponds to surgeries in children with CLO/CLP>1 y of age.

**‡**“Timely” corresponds to surgeries in children with CPO≤2 y of age; “late” corresponds to surgeries in children with CPO>2 y of age.

**Figure 1:**
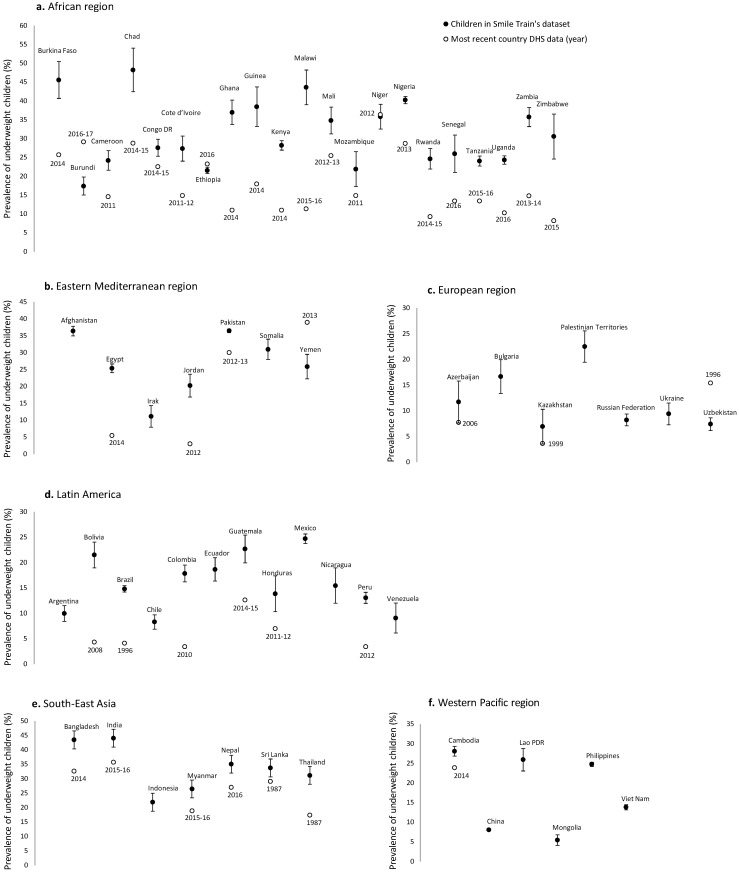
Prevalence of underweight in clinical records (in 60 countries) and in national surveys (available in 42 out of 60 countries).

**Figure 2:**
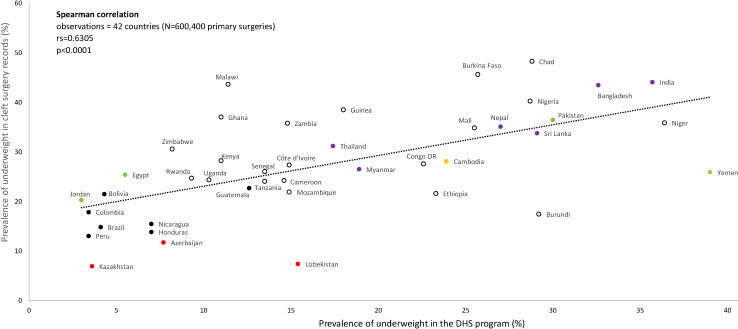
Correlation between the prevalence of underweight in clinical records and in national surveys across 42 countries. Spearman correlation: Observations = 42 countries (N = 600 400 primary surgeries) r_s_ = 0.6305. *P* < 0.0001.

We examined the prevalence of underweight by cleft types in children from 60 LMICs (Table S3 in the **Online Supplementary Document**). The prevalence of underweight among children at the time of surgery was either high (between 20%-29%) or very high (≥30%) in two thirds of all 60 countries (**Figure 1**). Comparisons with DHS data for 42 countries showed that, except for 5 countries (Burundi, Ethiopia, Niger, Yemen, and Uzbekistan), the prevalence of underweight was higher in children with cleft than in those without cleft (*P* < 0.05) (**Figure 1**). We also reported a positive correlation between the prevalence of underweight among children with cleft and the prevalence of underweight in the DHS program (r_s_ = 0.6305; *P* < 0.0001) (**Figure 2**). The correlation persisted when the analysis was restricted to either children with CLO (r_s_ = 0.5945, *P* < 0.0001), CPO (r_s_ = 0.5157, *P* < 0.001), or CLP (r_s_ = 0.5634, *P* = 0.0001).

## DISCUSSION

Orofacial cleft is not considered to be a life-threatening condition requiring emergency surgery. Nonetheless, the analysis of over 600 000 records of primary cleft operations conducted in low- and middle-income countries (LMICs) over a 10-year-period showed that 28.6% of children were underweight at the time of primary surgery. This figure is well above the global underweight prevalence in children ≤5 years estimated at 13.5% in 2017 [11] and reflects the additional unmet needs of children with cleft in LMICs.

To date, evidence of malnutrition in children with unrepaired cleft in limited-resource settings has come from small observational studies that described the nutritional status of patients using different anthropometric indices, different reference growth curves, and/or different cut-offs to evaluate the prevalence of malnutrition making it difficult to provide a global estimate [12-18]. Two studies described malnutrition using weight-for-age z scores with a cut-off of -2 SD using the NCHS reference growth curves as reference [12,14]. One study, led by Lazarus and colleagues [14] over two decades ago in South Africa, provided high prevalence estimates of underweight in 640 children with cleft at the time of primary surgery: 21.8% with CLO, 36.1% with CPO, and 29.6% with (unilateral) CLP. The other study conducted in Nigeria compared the nutritional status of 50 children with cleft and 50 peers without cleft [12]. The study reported that 26% of the children with cleft were underweight yet found no evidence of a difference with children without cleft. We showed however that, across limited-resource settings, malnutrition was more prevalent in children with a cleft than in their peers born without a cleft.

Countries with the highest prevalence estimates of underweight children in the general population are also those with the highest prevalence of underweight in children with cleft. This implies that a proportion of the risk of children with cleft to fail to thrive is attributable to setting-specific determinants of the nutritional situation of children in a country. However, our finding that underweight is more prevalent in children with cleft demonstrates that they face more challenges to thrive than their peers without cleft. The literature on the impact of the presence of a cleft on the care and safety of children in LMICs is however limited. Regardless of the setting, the risk of failure to thrive in newborns with cleft is inherent to the condition itself, as a cleft can immediately interfere with the ability for these children to feed. Feeding challenges are especially common in infants born with a cleft of the palate, with a bilateral cleft of the lip, and/or when there are associated anomalies [19]. Difficulty with creating a tight seal of the lips around the nipple and a hole in the palate both affect the normal sucking process, placing children at immediate risk of undernutrition and failure to thrive [20,21]. Nonetheless, feeding difficulties can be mitigated with timely and appropriate feeding assistance to the mother [22]. Yet, in many LMICs, children with cleft are unlikely to present at hospitals unless they have been actively searched for and identified in the community. Our data show that delays in accessing surgery cannot solely be responsible for children being found malnourished. Late presentations to cleft care teams may further delay access to appropriate nutrition care, but it is the lack of feeding assistance from the time of birth that plays a major role in driving malnutrition in this population. Besides, we found that the prevalence of underweight was high even in children with CLO although unilateral cleft lip is usually thought to have little impact on the feeding process [13,21]. Stigma due to misconceptions and ignorance around cleft is a major barrier to care for these children [23]. Social neglect may be especially affecting children with CL±P as the cleft of the lip is prominent and disfiguring. Additional challenges known to prevent timely and appropriate care of neonates and infants with disability in low-resource settings can also explain a higher prevalence of malnutrition among children with unrepaired cleft [24,25]. Barriers related to sociocultural norms and family structure, education, household income, distance to health facilities, or availability and cost of health care, may contribute, to various degrees, to the lack of or inadequate care provision to children with cleft. For newborns with CPO, the lack of palate examination and/or awareness of cleft can also result in missed opportunities to identify cases at the time of birth and to provide timely feeding support [4,26]. It is unclear why we found that, across most ethnic groups and settings, the prevalence of underweight was lowest among children with CPO at surgery, even when compared to children with CLP. Several reasons might explain these observations. Besides the struggle to feed, these children are at high risk of choking and death. CPO is also more likely to be associated with other congenital anomalies than CL±P, which may further threaten the survival of these children [27,28]. We thus hypothesised that those eventually accessing surgery might be among the mildest CPO cases, that is to say, those more likely to have survived the lack of appropriate attention and care [4]. Another reason why underweight is less prevalent in children with CPO at surgery may be related to the fact that CPO affects more females than males [29]. Indeed, in line with previous reports [30], we found that the prevalence of underweight was systematically lower in female cases overall and across all age groups, ethnic groups, and settings.

This study is the first to examine the scale of the malnutrition burden in children with cleft across lower-resource countries where immediate care at birth and access to cleft surgery are limited. As we pooled together data collected over a 10-year period, we did not account for the possibility of a decrease in the prevalence of underweight at surgery in more recent years. Indeed, with a rise in the provision of comprehensive care (including feeding and nutrition care) by cleft care providers in LMICs, it is possible that less children are being malnourished at the time of surgery at present compared to ten years ago. Yet, a focus on recent years only would have reduced the breadth of this study. With only about 20% of surgical records from countries outside Southeast Asia and the Western Pacific region (with India and China being the main drivers in number of surgeries), we would not have reached the minimum of 200 surgeries per country (which we chose arbitrarily as a cut-off) in many countries to be able to compare the data set to DHS data. Further, our overall and country-specific findings only account for children who have been operated by Smile Train’s partners. Children who have not been identified, those who have been lost to follow-up before being operated, and those operated by other surgical teams are not accounted for. However, although the records are limited to clinical data, the relative proportions of cleft types in this database are reasonably consistent with the current understanding of cleft epidemiology across ethnic groups and settings [29,31]. Figures may also underestimate the scale of malnutrition because a substantial proportion of the children operated by Smile Train’s partners may have received some level of feeding and nutritional care prior to surgery to promote weight gain, correct anaemia, and ensure fitness to surgery. Besides, although there are weight-for-age reference data for children up to 10 years of age, we only analysed records from children aged ≤5 years. Even though we have not examined the prevalence of underweight in older children (5-10 years), we can at best assume that, given that the data set includes only children who were deemed fit to undergo surgery by cleft surgical teams, an analysis would not have shown a greater prevalence of underweight in this older age group compared to younger children. Another potential limitation of the present study relates to the use of weight-for-age z scores to estimate the nutritional status of children with cleft. Weight-for-age is a composite index that cannot distinguish between different forms of malnutrition [32] and additional anthropometric indices – ie, height-for-age and weight-for-height – are needed to establish the prevalence of stunting and wasting [33]. Nonetheless, because it is a composite index, weight-for-age allows to consider all malnourished children – whether they are wasted, stunted, or both wasted and stunted. We cannot exclude that values for weight-for-age z scores are biased by inaccuracies in dates of birth and weight measurements [32]. However, weight is easy to measure such that measurements are less likely to be inaccurate than height measurements. Finally, the use of country-specific DHS estimates as an approximation of the nutritional situation of (non-cleft) children ≤5 years old may be limited by the fact that (i) survey data are captured at specific points in time, whereas we estimated the prevalence of underweight using clinical data collected over a 10-year period, (ii) the age distribution in our data set may not match the age distribution in DHS survey samples, and (iii) national estimates were not available for all countries and in some cases (eg, Sri Lanka) were not recent. Nonetheless, DHS data provided reliable country-specific estimates of underweight prevalence readily available for comparisons.

## CONCLUSIONS

Our findings highlight the urgent need of initiatives on child and maternal health that will equip parents, communities, birth attendants, midwives, nutritionists, and other health care professionals in LMICs with the necessary knowledge and skills that will ensure early identification of patients with cleft and prompt provision of feeding care. Barriers to early care provision need to be addressed to give children with cleft in LMICs the chance to survive and develop to their full potential. Efforts to protect the most disadvantaged children, including those born with a cleft, are paramount to achieve significant progress towards the health targets of SDG 3 [34] and concomitantly reduce health inequalities within countries.

## Additional material


Online Supplementary Document
