# Supplementary material for: Prevalence of malnutrition among children at primary cleft surgery: A cross-sectional analysis of a global database
Source: J Glob Health. 2022 Feb 26;12:04012. doi: 10.7189/jogh.12.04012 (PMC8874895; doi:10.7189/jogh.12.04012)
Supplement: Online Supplementary Document [file jogh-12-04012-s001.pdf]

Table S1. Geographic and ethnic distribution in cleft records (N=602,568).

WORLD REGIONS*	NUMBER OF COUNTRIES	COUNTRIES	ETHNIC GROUPS
**TOTAL**	**101**		
**African region**	37	Angola, Benin, Burkina Faso, Burundi, Cameroon, Central African Republic, Chad, Comoros, Congo DR, Cote d'Ivoire, Ethiopia, Gabon, Gambia, Ghana, Guinea, Guinea Bissau, Kenya, Liberia, Madagascar, Malawi, Mali, Mauritania, Mauritius, Mozambique, Niger, Nigeria, Republic of Congo, Rwanda , Senegal, Sierra Leone, South Africa, South Sudan, Tanzania, Togo, Uganda, Zambia, Zimbabwe	98.6% Black Africans<br>1.4% Other
**Eastern Mediterranean region**	15	Afghanistan, Djibouti, Egypt, Iraq, Jordan, Libya, Oman, Pakistan, Qatar, Saudi Arabia, Somalia, Sudan, Syria, United Arab Emirates, Yemen	68.8% Asians<br>17.2% Indians<br>14% Other
**European region**	15	Azerbaijan, Belarus, Bulgaria, Georgia, Kazakhstan, Kuwait, Kyrgyzstan, Lebanon, Lithuania, Palestinian Territories, Russian Federation, Tajikistan, Turkmenistan, Ukraine, Uzbekistan	57.4% Caucasians<br>32.1% Asians<br>10.5% Other
**Region of the Americas**	18	Argentina, Bolivia, Brazil, Chile, Colombia, Dominican Republic, Ecuador, El Salvador, Guatemala, Guyana, Haiti, Honduras, Mexico, Nicaragua, Panama, Paraguay, Peru, Venezuela	59.3% Hispanics<br>19.0% Caucasians<br>13.2% Mixed<br>8.5% Other
**South-East Asia region**	10	Bangladesh, Bhutan, Christmas Island, India, Indonesia, Maldives, Myanmar, Nepal, Sri Lanka, Thailand	73.1% Indians<br>26.1% Asians<br>0.8% Other
**Western Pacific region**	6	Cambodia, China, Lao PDR, Mongolia, Philippines, Viet Nam	99.9% Asians<br>0.1% Other

* According to WHO

Table S2. Median age at the time of surgery and prevalence of late primary operations across cleft types, sex, ethnicities, and world regions

	MEDIAN AGE AT SURGERY (Q_1,Q_3) IN YEARS			% LATE PRIMARY SURGERIES (95% CI)		
	CLO†	CPO†	CLP†	CLO (>1 year)	CPO (>2 years)	CLP (>1 year)
**TOTAL***	0.72 (0.45,1.55)	1.68 (1.10,2.82)	0.88 (0.51,1.60)	37.9 (37.6-38.1)	41.6 (41.3-41.9)	44.3 (44.1-44.4)
**Sex:**						
Male	0.71 (0.44,1.55)	1.66 (1.10,2.78)	0.90 (0.51,1.62)	37.8 (37.4-38.1)	40.9 (40.5-41.3)	44.9 (44.7-45.1)
Female	0.72 (0.45,1.55)	1.71 (1.10,2.86)	0.85 (0.50,1.56)	38.0 (37.6-38.5)	42.4 (42.0-42.8)	43.1 (42.8-43.4)
**Ethnic groups:**						
Asian	0.64 (0.42,1.29)	1.84 (1.21,2.96)	0.92 (0.50,1.71)	32.7 (32.3-33.1)	45.4 (45.1-45.8)	46.2 (46.0-46.5)
Black African	0.94 (0.47,2.20)	1.70 (1.03,2.87)	0.77 (0.42,1.64)	47.7 (46.9-46.5)	42.2 (40.7-43.8)	41.0 (40.4-41.5)
Indian	0.86 (0.51,1.98)	1.48 (1.01,2.56)	0.88 (0.53,1.52)	44.5 (44.0-45.0)	36.3 (35.8-36.8)	43.5 (43.2-43.7)
Hispanic	0.53 (0.36,0.97)	1.61 (1.10,2.45)	0.82 (0.43,1.48)	24.1 (22.1-26.1)	36.3 (34.4-38.3)	41.7 (41.0-42.5)
Caucasian	0.50 (0.35,0.82)	1.38 (1.03,2.15)	0.77 (0.43,1.36)	20.1 (17.9-22.4)	27.9 (26.1-29.7)	41.9 (40.8-43.1)
Mixed/Other	0.59 (0.35,1.35)	1.42 (0.97,2.35)	0.72 (0.39,1.36)	32.6 (30.6-34.7)	30.0 (28.1-31.9)	36.9 (35.8-38.0)
**World regions:**						
African Region	0.93 (0.47,2.18)	1.69 (1.03,2.84)	0.76 (0.41,1.62)	47.4 (46.6-48.2)	42.1 (40.5-43.7)	40.2 (39.6-40.8)
Eastern Mediterranean Region	0.74 (0.42,2.00)	1.49 (1.00,2.58)	0.84 (0.48,1.56)	40.3 (39.1-41.4)	36.5 (35.5-37.5)	41.8 (41.3-42.4)
European Region	0.58 (0.41,1.01)	1.69 (1.12,2.97)	0.85 (0.51,1.49)	25.4 (22.5-28.4)	40.5 (38.3-42.7)	42.9 (41.3-44.5)
Region of the Americas	0.53 (0.36,0.96)	1.56 (1.11,2.37)	0.82 (0.43,1.49)	23.6 (22.1-25.1)	33.3 (31.9-34.6)	42.6 (42.0-43.2)
South-East Asia Region	0.88 (0.52,1.99)	1.51 (1.02,2.61)	0.90 (0.53,1.57)	45.2 (44.8-45.7)	37.3 (36.8-37.8)	44.6 (44.4-44.8)
Western Pacific Region	0.59 (0.40,1.09)	1.89 (1.26,2.98)	0.90 (0.49,1.72)	27.7 (27.3-28.1)	46.8 (46.4-47.2)	46.1 (45.8-46.5)

* 602,568 cases

† CLO, Cleft lip only; CPO, Cleft palate only; CLP, Cleft lip and palate

Table S3. Prevalence of underweight at the time of primary cleft surgery in 60 countries*.

	COUNT	WEIGHT-FOR-AGE Z SCORE <-2 SD	COUNT	WEIGHT-FOR-AGE Z SCORE <-2 SD	COUNT	WEIGHT-FOR-AGE Z SCORE <-2 SD	COUNT	WEIGHT-FOR-AGE Z SCORE <-2 SD	COU[illegible]
**AFRICAN REGION:**	**Burkina Faso**		**Burundi**		**Cameroon**		**Chad**		
CLO	58	34.48	554	14.98	294	22.79	57	36.84	[illegible]
CPO	37	54.05	189	7.41	155	16.77	7	42.86	[illegible]
CLP	302	46.69	226	31.86	579	26.94	222	51.35	[illegible]
All clefts	397	45.59	969	17.44	1,028	24.22	286	48.25	[illegible]
DHS†		*25.70*		*29.20*		*14.60*		*28.80*	
	**Ghana**		**Guinea**		**Kenya**		**Malawi**		
CLO	139	27.34	42	26.19	1,702	23.15	74	14.86	[illegible]
CPO	149	31.54	29	13.79	408	24.02	42	38.10	[illegible]
CLP	569	40.77	259	43.24	2,706	32.04	331	50.76	[illegible]
All clefts	857	36.99	330	38.48	4,816	28.22	447	43.62	[illegible]
DHS†		*11.00*		*18.00*		*11.00*		*11.40*	
	**Nigeria**		**Rwanda**		**Senegal**		**Tanzania**		
CLO	2,765	34.90	273	15.38	68	19.12	1,345	23.94	[illegible]
CPO	815	26.01	108	18.52	38	39.47	303	26.40	[illegible]
CLP	6,236	44.47	567	30.34	194	25.77	2,349	23.80	[illegible]
All clefts	9,816	40.24	948	24.68	300	26.00	3,997	24.04	[illegible]
DHS†		*28.70*		*9.30*		*14.30*		*13.50*	
**EASTERN MEDITERRANEAN REGION:**	**Afghanistan**		**Egypt**		**Irak**		**Jordan**		
CLO	333	25.83	877	27.94	98	11.22	117	21.37	[illegible]
CPO	613	28.71	857	9.45	119	8.40	153	16.99	[illegible]
CLP	3,422	38.81	2,647	29.69	151	13.25	273	21.61	[illegible]
All clefts	4,368	36.40	4,381	25.38	368	11.14	543	20.26	[illegible]
DHS‡		_		*5.50*		_		*3.00*	

**EUROPEAN REGION:**	**Azerbaijan**		**Bulgaria**		**Kazakhstan**		**Palestinian Territories**		
CLO	30	6.67	64	6.25	30	10.00	134	26.87	[illegible]
CPO	70	11.43	130	15.38	37	5.41	213	11.27	[illegible]
CLP	139	12.95	304	19.41	150	6.67	373	27.35	[illegible]
All clefts	239	11.72	498	16.67	217	6.91	720	22.50	[illegible]
DHS§		*7.70*		_		*3.60*		_	
**REGION OF THE AMERICAS:**	**Argentina**		**Bolivia**		**Brazil**		**Chile**		
CLO	74	5.41	101	43.56	1,303	15.27	91	15.38	[illegible]
CPO	160	6.88	68	22.06	2,367	12.04	279	6.81	[illegible]
CLP	1,200	10.67	831	18.77	8,005	15.57	1,090	8.07	[illegible]
All clefts	1,434	9.97	1,000	21.50	11,675	14.82	1,460	8.29	[illegible]
DHS‖		_		*4.30*		*4.10*		_	
	**Honduras**		**Mexico**		**Nicaragua**		**Peru**		
CLO	35	5.71	656	23.17	22	18.18	339	8.26	[illegible]
CPO	45	6.67	792	16.04	48	8.33	393	7.12	[illegible]
CLP	288	15.97	6,807	25.86	344	16.28	2,993	14.37	[illegible]
All clefts	368	13.86	8,255	24.70	414	15.46	3,725	13.05	[illegible]
DHS‖		*7.00*		_		*7.40*		*3.40*	
**SOUTH-EAST ASIA REGION:**	**Bangladesh**		**India**		**Indonesia**		**Myanmar**		
CLO	4,687	40.54	32,003	44.84	7,640	21.10	621	23.83	[illegible]
CPO	4,068	34.81	34,008	33.07	3,734	18.34	332	27.71	[illegible]
CLP	17,348	46.29	140,664	46.56	27,023	22.57	2,555	26.97	[illegible]
All clefts	26,103	43.47	206,675	44.07	38,397	21.87	3,508	26.48	[illegible]
DHS¶		*32.60*		*35.70*		_		*18.90*	
**WESTERN PACIFIC REGION:**	**Cambodia**		**China**		**Lao PDR**		**Mongolia**		
CLO	431	23.43	35,930	8.17	199	23.12	86	2.33	[illegible]
CPO	1,260	24.29	46,182	6.11	158	28.48	180	3.89	[illegible]

CLP	3,269	30.19	60,372	9.52	545	26.24	814	6.14	19,0
All clefts	4,960	28.10	142,484	8.08	902	25.94	1,080	5.46	26,1
*DHS***		*23.90*		_		_		_	

* Countries with >200 total cleft cases (2,168 cases exluded). Excluded countries include: African region: Angola (N=8), Benin (N=178), Central African Republic (N=27), Comoros (N=3), Gabon (N=29), Gambia (N=139), Guinea Bissau Mauritania (N=153), Mauritius (N=2), Republic of Congo (N=198), Sierra Leone (N=101), South Africa (N=19), South Sudan (N=42), Togo (N=191); Eastern Mediterranean region: Djibouti (N=73), Libya (N=1), Oman (N=1), Qatar (N=3 United Arab Emirates (N=4); European region: Belarus (N=2), Georgia (N=90), Kuwait (N=1), Kyrgystan (N=4), Lebanon (N=34), Lithuania (N=2), Tajikistan (N=59), Turkmenistan (N=2); Region of the Americas: Dominican Republic (N Haiti (N=179), Panama (N=40), Paraguay (N=7); South-East Asia region: Bhutan (N=88), Christmas Island (N=1), Maldives (N=5).

† Most recently published data (as of December 2018). Burkina Faso: 2014; Burundi: 2016-17; Cameroon: 2011; Chad: 2014-15; Congo DR:2013-14; Cote d'Ivoire: 2011-12; Ethiopia: 2016; Ghana: 2014; Guinea: 2014; Kenya: 2014; Mala Niger: 2012; Nigeria: 2013; Rwanda: 2014-15; Senegal: 2017; Tanzania: 2015-16; Uganda: 2016; Zambia: 2013-14; Zimbabwe: 2015.

‡ Most recently published data. Egypt: 2014; Jordan: 2012; Pakistan: 2012-13; Yemen: 2013.

§ Most recently published data. Azerbaijan: 2006; Kazakhstan: 1999; Uzbekistan: 1996.

‖ Most recently published data. Bolivia: 2008; Brazil: 1996; Colombia: 2010; Guatemala: 2014-15; Honduras: 2011-12; Nicaragua: 2001; Peru: 2012.

¶ Most recently published data. Bangladesh: 2014; India: 2015-16; Myanmar: 2015-16; Nepal: 2016; Sri Lanka: 1987; Thailand: 1987.

** Most recently published data. Cambodia: 2014.
